# Bile acids differentially impact on platelet activation

**DOI:** 10.1186/1753-6561-6-S4-P22

**Published:** 2012-07-09

**Authors:** J Tan, E Reddy, D Murphy, S Keely, S O'Neill

**Affiliations:** 1 Royal College of Surgeons in Ireland, Ireland; 2 Molecular and Cellular Therapeutics, Royal College of Surgeons in Ireland, Ireland; 3 Molecular Medicine, Royal College of Surgeons in Ireland, Ireland

## Introduction

Platelets are well-established mediators of inflammatory responses throughout the body. Increased platelet activity occurs in the intestinal mucosa in conditions of Inflammatory Bowel Disease (IBD), where it may contribute to the mucosal fibrosis associated with these diseases. The bile acids, ursodeoxycholic acid (UDCA), deoxycholic acid (DCA) and tauroursodeoxycholic acid (TUDCA) were shown to regulate inflammatory responses in the intestine. The aim of this study is to analyse the impact of these bile acids on platelet activation.

## Methods

Gel-filtered platelets were isolated from human blood. They were incubated with bile acids as follows: UDCA (250mM, 175mM, 100mM, 10mM); DCA and TUDCA (500mM, 100mM, 10mM) for 10 minutes at 37°C before activation with thrombin (0.1U/ml) and collagen (38mg/ml). The effects of the bile acids on platelet aggregation were examined using light transmission platelet aggregometry. Bile acid effects on platelet ADP secretion were assessed by measuring the level of luminescence from a luciferin-luciferase 96 well-based assay. Effects of the bile acids on platelet morphology were examined by confocal microscopy. An MTT (3-(4,5-Dimethylthiazol-2-yl)-2,5-diphenyltetrazolium bromide)-based assay was used to assess the effects of the bile acids on platelet viability.

## Results

UDCA inhibited platelet aggregation and ADP secretion in platelets activated with collagen, but not thrombin, in a concentration-dependent manner (n=4, p<0.05). However, DCA and TUDCA inhibited platelet aggregation and ADP secretion in platelets activated with either agonist (n=4, p<0.05). Confocal microscopy revealed decreased adhesion of platelets to collagen with increasing bile acid concentrations. However, UDCA had no effect on the adhesion of thrombin (0.1U/ml)-activated platelets to collagen. On the other hand, the presence of DCA and TUDCA reduced the adhesion of thrombin-activated platelets to collagen. MTT assays showed no significant effect of the bile acids on platelet viability.

## Conclusions

While the effects of DCA and TUDCA appear to be more global on platelet activation, the effects of UDCA are more specific towards collagen activation of platelets. While the mechanisms involved remain unknown, these results indicate UDCA may specifically act as an antagonist to the collagen receptor, a2b1. Such actions of UDCA could have important implications for therapeutically reducing thrombotic/fibrotic events in patients with IBD.

